# Epiphyseal separation of the trochlea due to stress-throwing injury in a pediatric baseball player: A case report and review of the literature

**DOI:** 10.1016/j.tcr.2025.101187

**Published:** 2025-05-26

**Authors:** Yusuke Hattori, Yohei Kawaguchi, Yuji Joyo, Sanshiro Yasuma, Hiroo Shiraga, Hideki Okamoto, Hideki Murakami, Yuko Waguri-Nagaya

**Affiliations:** a Department of Orthopaedic Surgery, Nagoya City University Graduate School of Medical Sciences, Nagoya, Japan; b Department of Orthopaedic Surgery, Nagoya City University East Medical Center, Nagoya, Japan

**Keywords:** Distal humeral epiphyseal separation, Epiphyseal separation of trochlea, Stress-throwing injury, Open surgery

## Abstract

Distal humeral epiphyseal separation (DHES) is rare and sometimes difficult to diagnose by radiography. This report presents a rare case of an 8-year-old male baseball player with epiphyseal separation of the trochlea caused by a stress-throwing injury. The patient sustained an elbow injury after throwing a ball. Ultrasound and magnetic resonance imaging confirmed epiphyseal separation of the trochlea and avulsion fracture of the capitellum. Open surgery was performed using 1.5 mm diameter K-wires. After 5 weeks of immobilization, an active range of motion was allowed. Bone union was achieved 4 months after surgery. The patient returned to baseball without pain. Open surgery may be a suitable treatment option for DHES as children grow.

## Highlights

• Distal humeral epiphyseal separation is rare and sometimes difficult to diagnose.
• Rare case of epiphyseal separation due to stress-throwing injury in a child
• Open surgery may be a suitable treatment option for DHES as children grow.

## Introduction

Elbow fractures are the most common type of fracture in the pediatric population [1]. However, distal humeral epiphyseal separation (DHES) is a rare condition and can be challenging to diagnose using radiography [2,3]. DHES commonly occurs in children aged <2 years and can be misdiagnosed as elbow dislocation on radiographs [2,3]. The typical mechanisms of this injury include rotational forces, shear forces, and hyperextension, usually occurring during birth trauma or due to child abuse [3,4]. However, the case we encountered is different from common cases.

In this report, we present a rare case of epiphyseal separation of the trochlea caused by a stress-throwing injury in a pediatric baseball player, which was successfully treated with open surgery. To the best of our knowledge, no similar cases have been reported in the literature. Additionally, a literature review was conducted to provide an overview of this injury.

## Case presentation

**Fig. 1:**
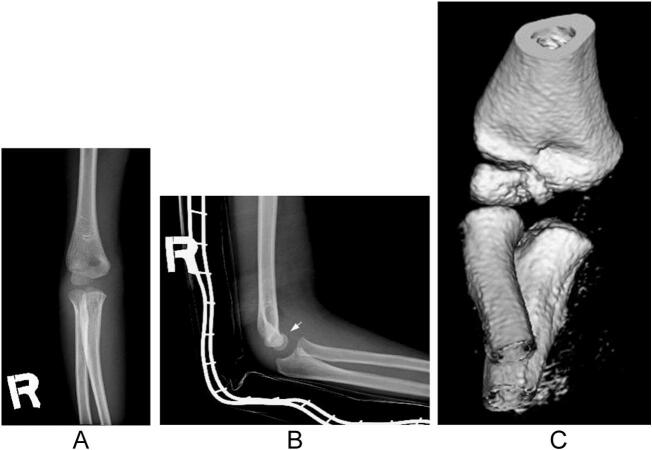
Simple X-ray and computed tomography (CT) images of the right elbow show an avulsion fracture of the capitellum. (a) Anteroposterior view. (b) Lateral view. The arrow indicates the avulsion fragment. (c) Three-dimensional anterior view.

**Fig. 2:**
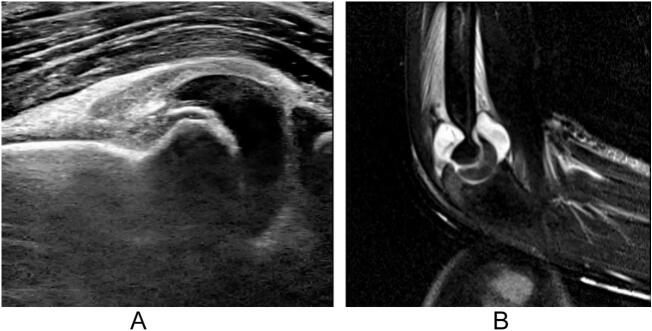
Ultrasound and magnetic resonance imaging (MRI) images show epiphyseal separation of the trochlea in the sagittal plane. (a) Ultrasound. (b) MRI.

An 8-year-old right-handed boy with a history of asthma was referred to our department with a painful injury in his right elbow. He played baseball twice a week for 2 years. During a three-day weekend, he played baseball for prolonged periods over three consecutive days. He sustained a painful injury to his right elbow after throwing the ball. On the same day, the patient promptly sought care at the emergency department of our hospital, and external immobilization with a splint was performed. The patient was referred to our department on the 2nd after injury. Clinical examination revealed swelling and tenderness of the right elbow. The range of motion (ROM) of the left elbow was 145°–20°, indicating joint laxity. Radiography revealed a small bony fragment anterior to the distal humerus on the lateral image (Fig. 1a and b). Computed tomography (CT) images showed an avulsion fracture of the capitellum (Fig. 1c). Ultrasound and magnetic resonance imaging (MRI) revealed epiphyseal separation of the trochlea in the sagittal plane (Fig. 2a and b). No other fractures or ligamentous injuries were observed. Written informed consent for treatment and publication of this case report was obtained from the patient's parents.

**Fig. 3:**
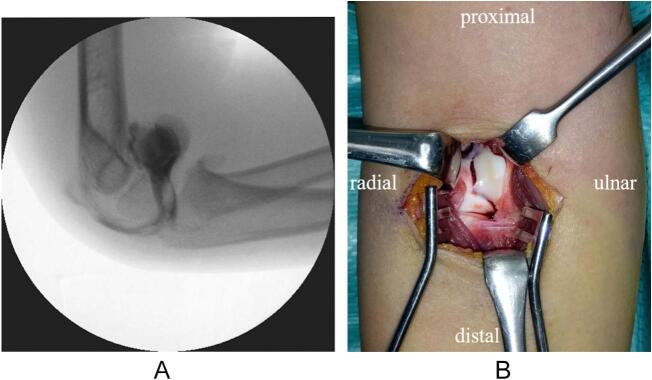
Intraoperative findings. (a) Arthrogram shows leakage of contrast agent anterior to the elbow, indicating an intraarticular fracture. (b) An intraoperative photograph of the fracture site using the anterior approach.

**Fig. 4:**
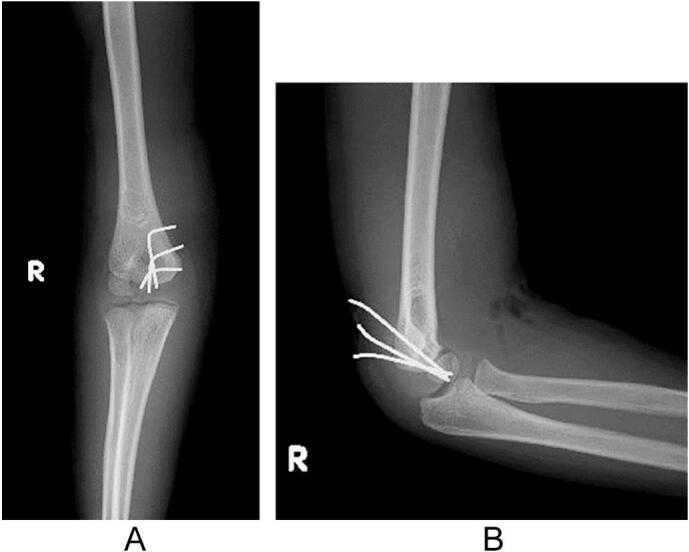
Postoperative plain X-ray of the right elbow. (a) Anteroposterior view. (b) Lateral view.

**Fig. 5:**
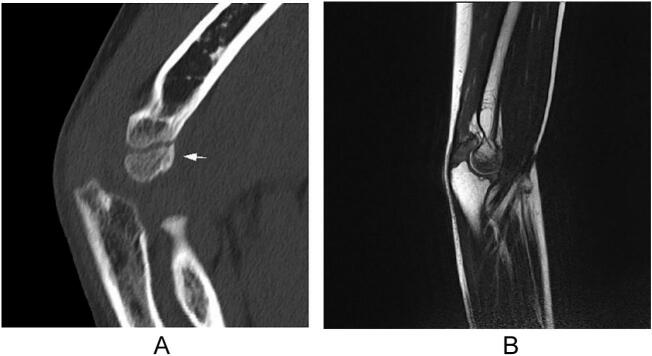
CT and MRI images at 4 months after surgery. (a) The arrow indicates the bone union site of the capitellum. (b) MRI shows a smooth articular congruity of the trochlea.

**Fig. 6:**
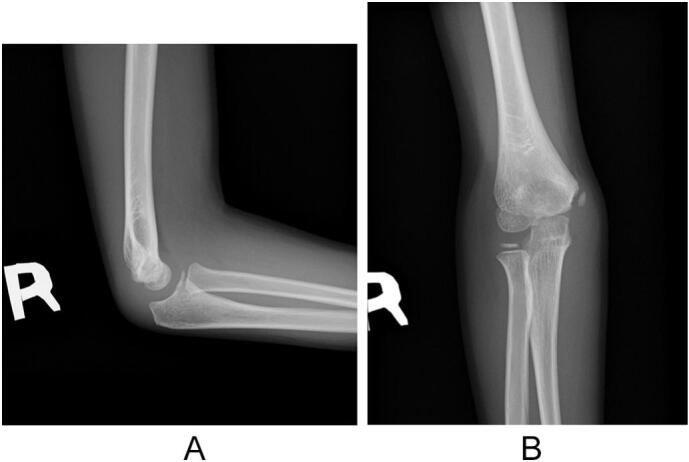
Plain X-ray images at 1 year after surgery. (a) Anteroposterior view. (b) Lateral view.

Arthrography of the right elbow was performed under general anesthesia on the 4th day after injury and revealed leakage of contrast agent anterior to the elbow, indicating an intraarticular fracture (Fig. 3a). Surgical treatment using the anterior approach was performed under tourniquet control. After the capsule was incised, a chondral fragment of the trochlea was found (Fig. 3b). The fragment was reduced and fixed with three 1.5 mm diameter K-wires (Fig. 4a and b). The elbow was immobilized with a cast in a 90° flexed position for 4 weeks, followed by a splint for 1 week. All wires were removed on an outpatient basis 4 weeks after surgery. Active ROM was allowed 5 weeks after surgery. CT scan was performed 4 months after surgery and revealed bone union of the capitellum (Fig. 5a). MRI revealed a smooth articular congruity of the trochlea, with no signs of bony necrosis (Fig. 5b). The patient did not feel any pain and returned to baseball. After 1 year of follow-up, the ROM for the right and left elbows was as follows: elbow flexion, 140° and 145°; elbow extension, 10° and 20°; forearm pronation, 90° bilaterally; and forearm supination, 100° bilaterally. The carrying angle was 5° bilaterally. The Baumann angle was 75° for the right elbow and 73° for the left elbow on radiographs (Fig. 6a and b).

## Discussion

DHES is a rare injury, accounting for 1.6 % of all distal humerus fractures in children [4]. This injury predominantly occurs in young children because the physeal cartilage at the junction of the layer of cartilage hypertrophy and primary bone spongiosa is more fragile than the bone [3]. Falling on an outstretched hand and traction and rotational forces are typical causes of DHES [3]. Regarding the direction of displacement, the distal fragment is usually displaced posteromedially and rarely posterolaterally [4]. DHES is frequently misdiagnosed. Supakul et al. reported that among 16 patients with DHES, DHES was misdiagnosed using radiographs in 9 (56 %) [3]. Therefore, ultrasound, MRI, and arthrography are important for definitive diagnosis.

This was a case of stress-throwing injury in an immature baseball player. Throwing injuries are sometimes problematic in children due to bone plasticity, ligamentous laxity, open epiphyseal growth plates, and underdeveloped musculature [5]. Improper throwing mechanics, excessive exposure, and pitching while fatigued are risk factors for throwing injuries, regardless of age [5]. Our case showed joint laxity and experienced fatigue from playing baseball consecutive, which is a risk factor for injury. DHES has been reported in young athletes [6,7]. However, these cases are classified as a Salter–Harris type I facture. In contrast, our case involved epiphyseal separation of the trochlea, indicating a Salter–Harris type III facture. Additionally, the distal fragment was displaced anteriorly. Thus, this case is notably different from previously reported cases.

The treatment of DHES remains controversial. Conservative treatment of DHES has proven effective in newborns and young children. However, complications, particularly cubitus varus, remain a concern [8,9]. Furthermore, closed reduction and pinning under an arthrogram are effective in clearly visualizing DHES [10]. However, this method has only been reported in young children. Chen et al. reported favorable outcomes of open anatomical reduction via the anterior mini-transverse approach [2]. Since our patient was older than typical patients, achieving the exact anatomical position was necessary. Additionally, the arthrogram images were insufficient to confirm fracture reduction. Therefore, open surgery was performed. The postoperative course was uneventful, and the patient eventually returned to baseball. Open surgery may be a viable treatment option for DHES as children grow.

## Conclusion

We reported a case of epiphyseal separation of the trochlea associated with stress-throwing injury treated with open surgery. Open surgery may be an appropriate treatment option for children with DHES as they grow.

## Statement of informed consent

The patient's parents provided written informed consent for publishing this case.

## CRediT authorship contribution statement

**Yusuke Hattori:** Investigation, Writing – original draft. **Yohei Kawaguchi:** Conceptualization, Methodology. **Yuji Joyo:** Project administration, Visualization. **Sanshiro Yasuma:** Writing – review & editing. **Hiroo Shiraga:** Writing – review & editing. **Hideki Okamoto:** Validation. **Hideki Murakami:** Writing – review & editing. **Yuko Waguri-Nagaya:** Supervision, Writing – review & editing.

## Funding

This study was partially supported by a Grant-in-Aid for Scientific Research (23K15746) from the Ministry of Education, Culture, Sports, Science, and Technology of Japan.

## Declaration of competing interest

The authors of this work have nothing to disclose.

## Data Availability

All research data are available in this article.
